# Power Pattern Sensitivity to Calibration Errors and Mutual Coupling in Linear Arrays through Circular Interval Arithmetics

**DOI:** 10.3390/s16060791

**Published:** 2016-05-31

**Authors:** Nicola Anselmi, Marco Salucci, Paolo Rocca, Andrea Massa

**Affiliations:** 1 ELEDIA@UniTN - University of Trento, Via Sommarive 9, I-38123 Trento, Italy; nicola.anselmi@eledia.org (N.A.); marco.salucci@eledia.org (M.S.); andrea.massa@eledia.org (A.M.); 2 ELEDIA@L2S - Laboratoire des Signaux et Systèmes, UMR8506 (CNRS - CS - UPS), 3 rue Joliot-Curie, 91192 Gif-sur-Yvette, France

**Keywords:** antenna arrays, linear arrays, sensitivity analysis, calibration errors, mutual coupling, interval analysis, circular intervals

## Abstract

The sensitivity to both calibration errors and mutual coupling effects of the power pattern radiated by a linear array is addressed. Starting from the knowledge of the nominal excitations of the array elements and the maximum uncertainty on their amplitudes, the bounds of the pattern deviations from the ideal one are analytically derived by exploiting the Circular Interval Analysis (CIA). A set of representative numerical results is reported and discussed to assess the effectiveness and the reliability of the proposed approach also in comparison with state-of-the-art methods and full-wave simulations.

## 1. Introduction

Phased arrays [1] are complex antenna systems whose implementation is a multi-step process starting from a preliminary design aimed at setting the main antenna characteristics (e.g., the size and the ideal current distribution) complying the project requirements, then followed by a detailed synthesis of the architecture, the feeding network, and the control logic before proceeding towards the prototype fabrication and its experimental characterization. The need to shorten the time-to-market and the ever increasing request of high-performance in current applications today (e.g., radars and communications) are pushing the development of reliable and robust analysis and synthesis tools able to predict (for defining suitable countermeasures or choosing alternative architectural solutions) the impacts on the actual radiated pattern of uncertainties and/or fabrication errors caused by the non-correspondence of the antenna model with its real implementation, thus minimizing expensive tuning procedures or re-design steps.

Originally, phased array synthesis methods [2,3] considered error-free isotropic elements to determine the values of the element excitations (*i.e.*, amplitudes and phases) generating the desired beam pattern. However, fabrication and calibration errors, uncertainties, and mutual coupling (MC) effects are present in real systems, and they unavoidably cause non-negligible deviations from the ideal antenna behavior. In order to predict these, several methodologies *a priori* modeling the effects of mechanical or manufacturing errors [4,5,6,7] as well as the MC effects [8,9,10] have been proposed. A common strategy is that of computing the configuration of the actual excitation weights as the product between the ideal ones and the coupling matrix [11], the main issue to be addressed being the accurate definition of the coupling model. Towards this aim, very accurate numerical techniques based on the method of moments have been presented [12,13], but unfortunately they generally turn out to be computationally expensive and usually they require customization to each type of array element. To define more general and efficient tools, even though less reliable, approximated methods have been also introduced in [14,15] where the currents induced on the array elements by the coupling effects are computed through analytic relationships. Recently, an alternative strategy has been described in [16] aimed at predicting the worst-case bounds of the radiated beam pattern by exploiting the Cauchy–Schwartz (CS) inequality and starting from the knowledge of the calibration errors (*i.e.*, the tolerances on the excitations due to the manufacturing imperfections of the devices composing the feeding network) and the coupling coefficients modeling the effects of the energy interchange among neighboring array elements.

Dealing with calibration errors, it is also worth mentioning a method based on Interval Analysis (IA) [17] devoted to determining the bounds of the deviations from the ideal/nominal power pattern in the case of both discrete (*i.e.*, antenna arrays [18,19,20,21,22]) and continuous (*i.e.*, reflector antennas [23,24]) apertures. The IA is a mathematical framework based on a set of arithmetic rules and properties, which allows operation and function evaluations when the arguments are intervals instead of crisp values, just involving the endpoints of the interval arguments and thus minimizing the computational costs to yield reliable and robust interval bounds as guaranteed by the *Inclusion Theorem* of IA [17,25]. Therefore, IA proved to be very suitable in all problems where physical quantities (e.g., uncertainties, errors, and irregularities) are modeled as intervals of unknown/random deviations from a nominal value.

In this work, an innovative IA-based approach to the sensitivity analysis of transmit antenna arrays in the presence of calibration errors and MC effects, as in [16], is presented. The circular version of the IA (CIA) [26] is exploited here for the first time to the best of the authors’ knowledge, since it is more adaptable for dealing with the effects on the pattern bounds of both amplitude and phase errors in the array excitations than the Cartesian IA (hereinafter called rectangular IA-RIA) used in previous works [18,19,20,21,23,24].

The outline of the paper is as follows. The problem is mathematically formulated in Section 2 where the analytic expressions of the dependence of the pattern bounds on the calibration uncertainties and mutual coupling effects are determined by means of the CIA-based approach, as well. A set of representative numerical results is reported in Section 3 to illustrate the behavior and the performance of the proposed sensitivity tool also in comparison with state-of-the-art methods (Section 3.1) and the results from a full-wave commercial solver (Section 3.2). Eventually, some conclusions are drawn (Section 4).

## 2. Mathematical Formulation

Let us consider a linear antenna array of *N* isotropic elements uniformly-spaced (*d* being the inter-element distance) along the *x*-axis. In an ideal case (*i.e.*, isotropic radiators without errors on the excitation weights), the nominal array factor is mathematically expressed as a function of the observation angle $\theta \in \left[-\frac{\pi }{2};\;\frac{\pi }{2}\right]$ as [1,3]:

(1) $$AF_{Nom}\left(\theta \right)=\frac{1}{\chi }\sum_{n=1}^{N}w_{n}e^{jk(n-1)dsin\theta }$$

where $w_{n}$, n=1,...,N is the set of the nominal (amplitude and phase) weights, $k=\frac{2\pi }{\lambda }$ is the free space wavenumber, *λ* being the wavelength, and $\chi =max_{\theta }\left\{\left|AF_{Nom}\left(\theta \right)\right|\right\}=\sum_{n=1}^{N}\left|w_{n}\right|$ is the normalization coefficient.

Dealing with real antennas, the arising calibration errors and mutual coupling effects can be modeled as amplitude and phase uncertainties on the excitations whose values turn out to be

(2) $$\underline{\tilde{w}}={\underline{w}}^{T}\left(\underline{\underline{C}}+\underline{\underline{I}}\right)$$

where $\underline{w}=\left\{w_{n}:\;n=1,...,N\right\}$, $\underline{\underline{I}}$ is the identity matrix, *T* denotes the transpose operation, and $\underline{\underline{C}}$ is a complex-valued matrix of dimensions N×N, function of the scan angle [1,3], whose entries along the principal diagonal, $c_{n,n}≜\gamma_{n}$, n=1,...,N, are the calibration error coefficients, while the others are the mutual coupling terms, $c_{i,j}≜\xi_{i,j}$, i,j=1,...,N, i≠j [16]. Then, the actual expression of the array factor is given by

(3) $$AF_{Act}\left(\theta \right)=\frac{1}{\chi }\sum_{n=1}^{N}{\tilde{w}}_{n}e^{jk(n-1)dsin\theta }\;$$

**Figure 1:**
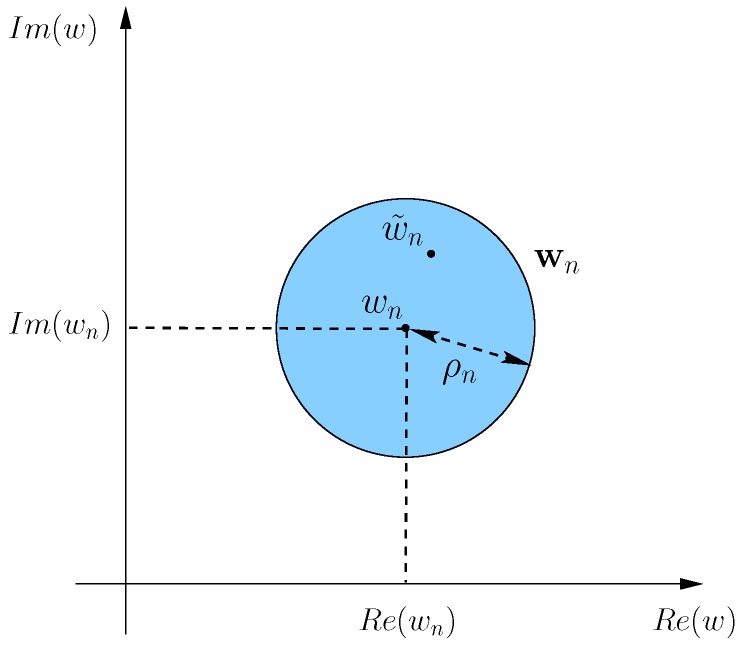
Interval Analysis (IA)-based approach—complex circular interval.

By considering the amplitude and the phase deviations from the nominal excitations ($w_{n}$, n=1,...,N) due to the calibration errors and mutual coupling effects both quantified by complex coefficients (*i.e.*, $c_{i,j}$, i,j=1,...,N), let us model the mathematical uncertainty on the knowledge of the *n*-th array element excitation as a circle in the complex plane centered at $w_{n}$ with radius $\rho_{n}$ (Figure 1). Such a circle encloses all possible values that the *n*-th (n=1,...,N) actual weight ${\tilde{w}}_{n}$ can assume regardless of the exact knowledge of the phase and the amplitude errors, while assuming a maximum amplitude uncertainty $\rho_{n}$ equal to [16]

(4) $$\rho_{n}=\left|\gamma_{n}\right|+\sum_{j=1,j\neq n}^{N}\left|\xi_{n,j}\right|\;,\;\;n=1,\ldots ,N\;$$

In an effective and compact representation, the whole set of admissible actual excitations can be represented as circular intervals (Appendix A) [26],

(5) $$w_{n}=\langle w_{n};\;\rho_{n}\rangle \;,\;\;n=1,\ldots ,N$$

univocally described by their barycenters, $w_{n}$, n=1,...,N (*i.e.*, the nominal coefficients), and radii, $\rho_{n}$, n=1,...,N (*i.e.*, the maximum amplitude errors). By substituting Equation (5) into Equation (3), the interval array factor for a given *θ* turns out to be:

(6) $$\mathrm{AF}\left(\theta \right)=\frac{1}{\chi }\sum_{n=1}^{N}w_{n}e^{jk(n-1)dsin\theta }$$

the sum of *N* circular intervals, each one given by

(7) $${\mathrm{AF}}_{n}\left(\theta \right)=w_{n}e^{jk(n-1)dsin\theta }$$

Since Equation (7) is the product of a circular interval $w_{n}$ to a complex value (*i.e.*, the exponential term), its explicit expression is determined according to Appendix B1

(8) $$\begin{array}{ccc} {\mathrm{AF}}_{n}\left(\theta \right) & = & \langle w_{n}e^{jk(n-1)dsin\theta };\;\left|e^{jk(n-1)dsin\theta }\right|\rho_{n}\rangle \\ & = & \langle w_{n}e^{jk(n-1)dsin\theta };\;\rho_{n}\rangle \; \end{array}$$

and the interval array factor in Equation (6) results in (Appendix B2)

(9) $$\begin{array}{ccc} \mathrm{AF}\left(\theta \right) & = & \frac{1}{\chi }\sum_{n=1}^{N}{\mathrm{AF}}_{n}\left(\theta \right)\\ & = & \frac{1}{\chi }\sum_{n=1}^{N}\langle w_{n}e^{jk(n-1)dsin\theta };\;\rho_{n}\rangle \\ & = & \langle \frac{1}{\chi }\sum_{n=1}^{N}w_{n}e^{jk(n-1)dsin\theta };\;\frac{1}{\chi }\sum_{n=1}^{N}\rho_{n}\rangle \end{array}$$

whose expression through Equation (1) is

(10) $$\mathrm{AF}\left(\theta \right)=\langle AF_{Nom}\left(\theta \right);\;\frac{1}{\chi }\sum_{n=1}^{N}\rho_{n}\rangle$$

As it can be noticed from Equation (10), the interval function $\mathrm{AF}\left(\theta \right)$, $\theta \in \left[-\frac{\pi }{2};\;\frac{\pi }{2}\right]$ is a complex-valued circular interval of center $AF_{Nom}\left(\theta \right)$ and radius equal to the maximum amplitude uncertainty, $\sum_{n=1}^{N}\rho_{n}$.

In order to determine the analytic expression of the interval power pattern, $P\left(\theta \right)≜{\left|\mathrm{AF}\left(\theta \right)\right|}^{2}$, the module of Equation (10) has to be firstly defined. By exploiting Appendix B3, the latter is equal to

(11) $$\left|\mathrm{AF}\left(\theta \right)\right|=\left[{\left|AF\left(\theta \right)\right|}_{Inf};\;{\left|AF\left(\theta \right)\right|}_{Sup}\right]$$

where

(12) $${\left|AF\left(\theta \right)\right|}_{Inf}=\max\left\{\left|AF_{Nom}\left(\theta \right)\right|-\frac{1}{\chi }\sum_{n=1}^{N}\rho_{n};\;0\right\}$$

and

(13) $${\left|AF\left(\theta \right)\right|}_{Sup}=\left|AF_{Nom}\left(\theta \right)\right|+\frac{1}{\chi }\sum_{n=1}^{N}\rho_{n}\;$$

Finally, $P\left(\theta \right)=\left[P_{Inf}\left(\theta \right);\;P_{Sup}\left(\theta \right)\right]$ is yielded by determining the analytic expressions of its bounds as a function of the nominal array factor, $AF_{Nom}\left(\theta \right)$, and the uncertainty values $\rho_{n}$, n=1,...,N. More specifically,

(14) $$P_{Sup}\left(\theta \right)={\left|AF_{Nom}\left(\theta \right)\right|}^{2}+{\left(\frac{1}{\chi }\sum_{n=1}^{N}\rho_{n}\right)}^{2}+\frac{2}{\chi }\left|AF_{Nom}\left(\theta \right)\right|\sum_{n=1}^{N}\rho_{n}$$

and if $\left|AF_{Nom}\left(\theta \right)\right|>\frac{1}{\chi }\sum_{n=1}^{N}\rho_{n}$, then

(15) $$P_{Inf}\left(\theta \right)={\left|AF_{Nom}\left(\theta \right)\right|}^{2}+{\left(\frac{1}{\chi }\sum_{n=1}^{N}\rho_{n}\right)}^{2}-\frac{2}{\chi }\left|AF_{Nom}\left(\theta \right)\right|\sum_{n=1}^{N}\rho_{n}$$

otherwise

(16) $$P_{Inf}\left(\theta \right)=0$$

## 3. Numerical Results

This section is devoted to the numerical validation of the proposed CIA-based sensitivity analysis tool. Representative results will illustrate the behavior of the CIA in evaluating the impact of calibration errors and mutual coupling effects (both modeled as amplitude and phase deviations of the complex excitation coefficients) on the radiated power pattern. Moreover, the reliability and the effectiveness of the proposed approach will also be analyzed through a comparative assessment carried out taking into account competitive state-of-the art approaches, namely the method recently proposed in [16] and based on the Cauchy-Schwartz inequality, as well as the rectangular version of the IA. Furthermore, the predictions from the CIA technique will be evaluated with respect to the full-wave simulation of a real array performed with a commercial software.

### 3.1. Validation and Comparative Assessment

**Figure 2:**
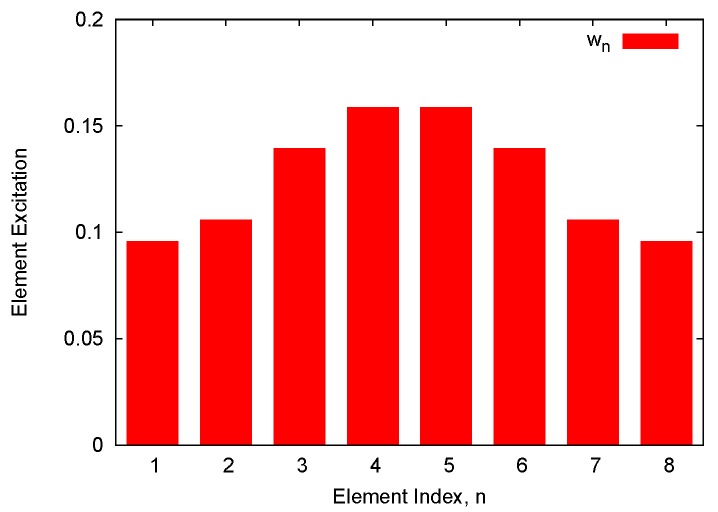
Performance Analysis (N=8, $d=\frac{\lambda }{2}$; Dolph–Chebyshev pattern: $SLL_{ref}=-20\;$dB)—Amplitude of the nominal excitations.

**Figure 3:**
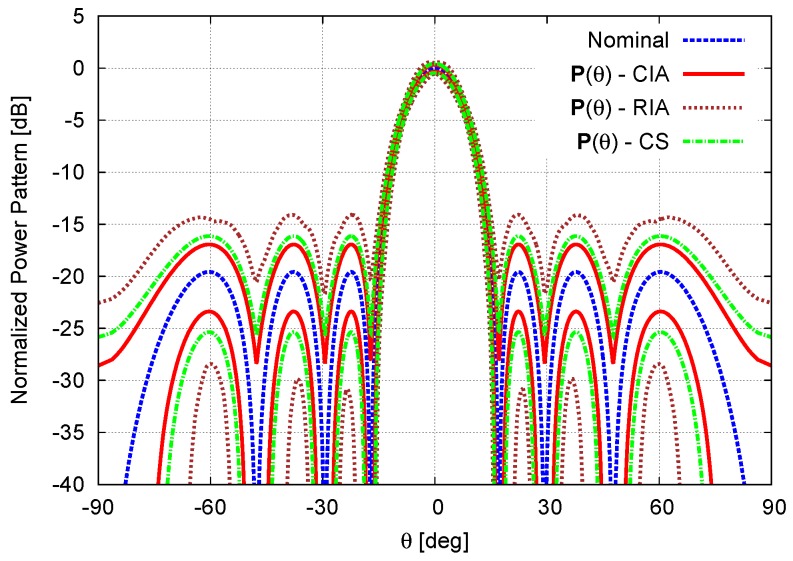
Calibration Error (N=8, $d=\frac{\lambda }{2}$; Dolph–Chebyshev pattern: $SLL_{ref}=-20\;$dB)—Nominal power pattern and interval power pattern bounds predicted by the the circular IA (CIA), the rectangular IA (RIA), and the Cauchy-Schwartz-based method (CS) [16].

**Figure 4:**
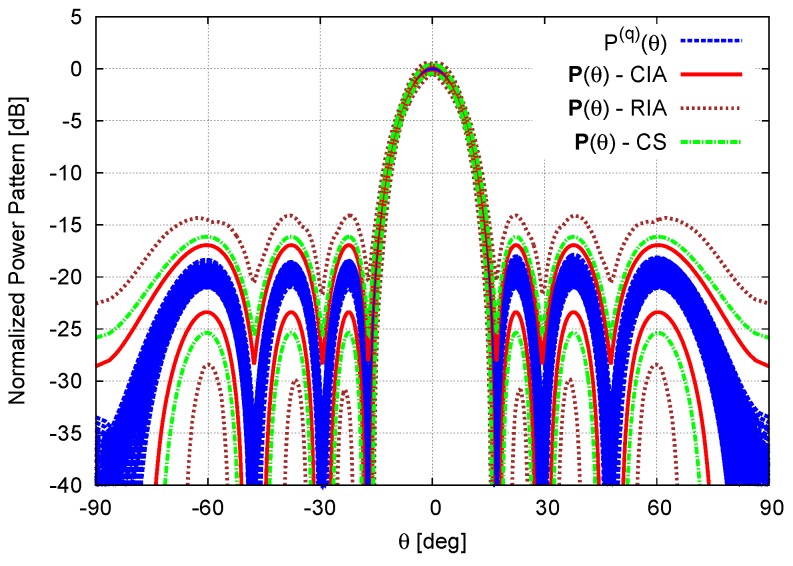
Calibration Error (N=8, $d=\frac{\lambda }{2}$; Dolph–Chebyshev pattern: $SLL_{ref}=-20\;$dB)—Plot of $Q=10^{5}$ Monte Carlo power patterns, $P^{q}\left(\theta \right)$, q=1,...,Q, along with the interval power pattern bounds as computed by the CIA, the RIA , and the CS [16].

As a benchmark test case, let us consider a linear antenna array made of N=8 isotropic elements equally-spaced by $d=\frac{\lambda }{2}$. The nominal excitations $w_{n}$, n=1,...,N, shown in Figure 2 and reported in Table 1, have been chosen to afford a Dolph–Chebyshev pattern with a side lobe level equal to $SLL_{ref}=-20\;dB$ [1,3]. The first example considers the array affected by calibration errors (*i.e.*, $c_{i,j}=0$, ∀i,j=1,...,N, i≠j) of values given as a percentage, $c_{n,n}=\gamma_{n}$, of the corresponding nominal excitation (Table 1—Calibration Error). Figure 3 shows the bounds of the interval power pattern $P\left(\theta \right)$ predicted with the CIA-based approach, the CS-based method [16], and the RIA along with the nominal plot. As a first consistency check, let us notice that whatever the method (CIA, CS, and RIA) the arising bounds include the nominal pattern. Moreover, the CIA bounds turn out to be tighter than those from the RIA (Indeed, the RIA excitations intervals, $w_{n}^{RIA}$, n=1,...,N are by definition the smallest complex-valued rectangles enclosing the corresponding circular intervals Equation (5), namely $w_{n}^{CIA}\in w_{n}^{RIA}$. Because of the *Inclusion* property of IA [17,25], the same condition holds true for the corresponding power patterns, $P{\left(\theta \right)}^{CIA}\in P{\left(\theta \right)}^{RIA}$), as expected, but also narrower than those from the CS ($P{\left(\theta \right)}_{Sup}^{CIA}\leq P{\left(\theta \right)}_{Sup}^{CS}\leq P{\left(\theta \right)}_{Sup}^{RIA}$ and $P{\left(\theta \right)}_{Inf}^{CIA}\geq P{\left(\theta \right)}_{Inf}^{CS}\geq P{\left(\theta \right)}_{Inf}^{RIA}$, $\theta \in \left[-\frac{\pi }{2};\;\frac{\pi }{2}\right]$). Of course, if the bound width is an index of the effectiveness of the prediction, on the other hand, it is mandatory that the bounds are inclusive (*i.e.*, $P^{q}\left(\theta \right)\in P\left(\theta \right)$ ∀q being $P^{q}\left(\theta \right)≜{\left|AF_{Act}^{q}\left(\theta \right)\right|}^{2}$ the power pattern radiated from the *q*-th setup of the actual excitations, $\forall {\tilde{w}}_{n}^{q}\in w_{n}$, n=1,...,N). In order to give an insight (An exhaustive proof of the inclusion property is unfeasible because of the need of generating the power patterns radiated by the infinite number of combinations of the actual excitations) on the reliability and the inclusiveness of CIA predictions, besides the theoretical support from IA *Inclusion Theorem* [17,25], $Q=10^{5}$ power patterns have been generated by randomly choosing ${\tilde{w}}_{n}^{q}$, n=1,...,N within the corresponding circular intervals $w_{n}$, n=1,...,N. Figure 4 confirms that all *Q* patterns lay within the CIA bounds.

**Figure 5:**
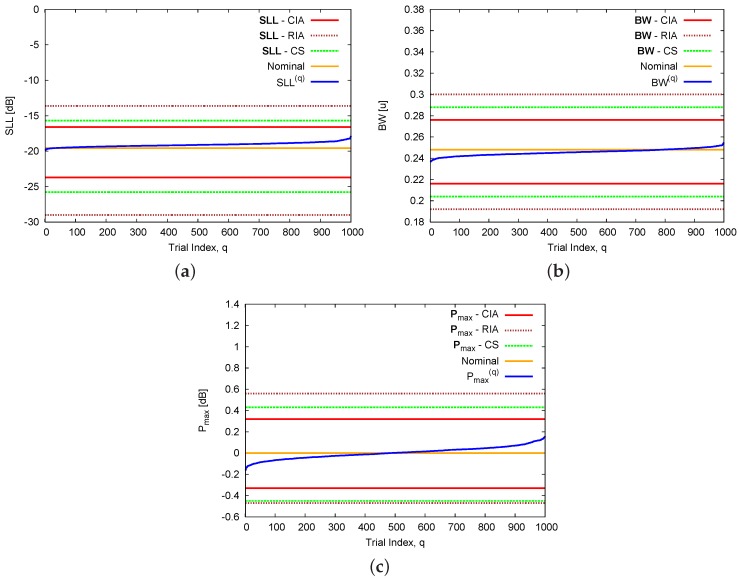
Calibration Error (N=8, $d=\frac{\lambda }{2}$; Dolph–Chebyshev pattern: $SLL_{ref}=-20\;$ dB)—Nominal values of the pattern indexes and bounds of the intervals (**a**) SLL; (**b**) BW; and (**c**) $P_{max}$ as computed by the CIA, the RIA , and the CS [16].

As for the pattern features, Figure 5 plots the endpoints (*i.e.*, lower and upper bounds) of the interval extensions of the sidelobe level (*i.e.*, SLL—Figure 5a) [18], the half-power beamwidth (*i.e.*, BW—Figure 5b) [18], and the peak power (*i.e.*, $P_{max}$—Figure 5c) [23] along with the corresponding nominal features as well as those of the *Q* randomly generated power patterns sorted in ascending order. As it can be observed (Figure 5) and quantitatively assessed (Table 2—Calibration Error), both the nominal and the *Q* samples are within their interval counterparts, while the CIA bounds are always contained within those of the CS that in turn lie within the RIA ones, namely $\psi_{Inf}^{RIA}\geq \psi_{Inf}^{CS}\geq \psi_{Inf}^{CIA}$ and $\psi_{Sup}^{CIA}\leq \psi_{Sup}^{CS}\leq \psi_{Sup}^{RIA}$ with $\psi =\left\{SLL,\;BW,\;P_{max}\right\}$. More in detail, the accuracy on the prediction, namely the interval width $\omega \left(\psi \right)≜\psi_{Sup}-\psi_{Inf}$, turns out to be improved by more than 29% ($\omega^{CIA}\left(\mathrm{SLL}\right)=7.10$ dB *vs.* $\omega^{CS}\left(\mathrm{SLL}\right)=10.11$ dB and $\omega^{RIA}\left(\mathrm{SLL}\right)=15.38$ dB), 28% ($\omega^{CIA}\left(\mathrm{BW}\right)=0.060\;\left[u\right]$ *vs.* $\omega^{CS}\left(\mathrm{BW}\right)=0.084\;\left[u\right]$ and $\omega^{RIA}\left(\mathrm{BW}\right)=0.108\;\left[u\right]$), and 26% ($\omega^{CIA}\left(P_{max}\right)=0.65$ dB *vs.* $\omega^{CS}\left(P_{max}\right)=0.88$ dB and $\omega^{RIA}\left(P_{max}\right)=1.03$ dB) for the sidelobe level (Table 2 – Figure 5a), the half-power beamwidth (Table 2 – Figure 5b), and the peak power (Table 2 – Figure 5c), respectively.

To further highlight the accuracy of the CIA sensitivity analysis, the metric of the *pattern tolerance index* △ [18]

(17) $$△=\frac{\int_{-\frac{\pi }{2}}^{\frac{\pi }{2}}\left(P{\left(\theta \right)}_{Sup}-P{\left(\theta \right)}_{Inf}\right)d\theta }{\int_{-\frac{\pi }{2}}^{\frac{\pi }{2}}{\left|AF_{Nom}\left(\theta \right)\right|}^{2}d\theta }$$

has been evaluated. It turns out that the improvement in the power pattern prediction of the CIA with respect to that from the CS and the RIA amounts to 27% and 57% (${△}^{CIA}=0.1493$ *vs.* ${△}^{CS}=0.2050$ and ${△}^{RIA}=0.3487$), respectively.

**Figure 6:**
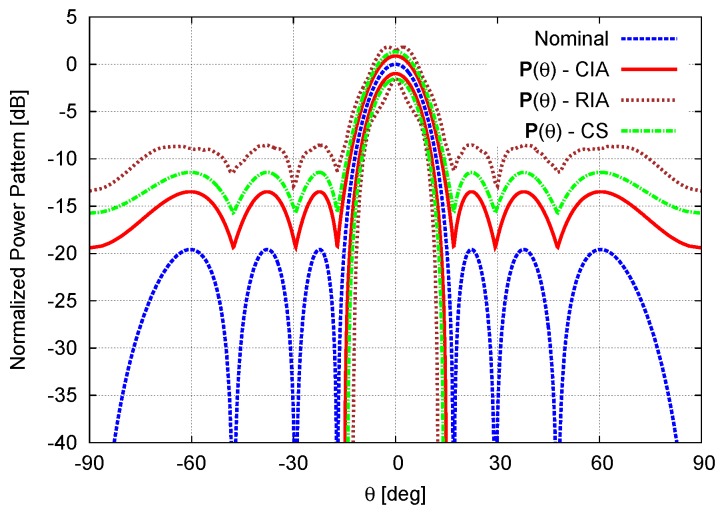
Adjacent Mutual Coupling (N=8, $d=\frac{\lambda }{2}$; Dolph–Chebyshev pattern: $SLL_{ref}=-20\;$dB)—Nominal power pattern and interval power pattern bounds predicted by the CIA, the RIA, and the CS [16].

**Figure 7:**
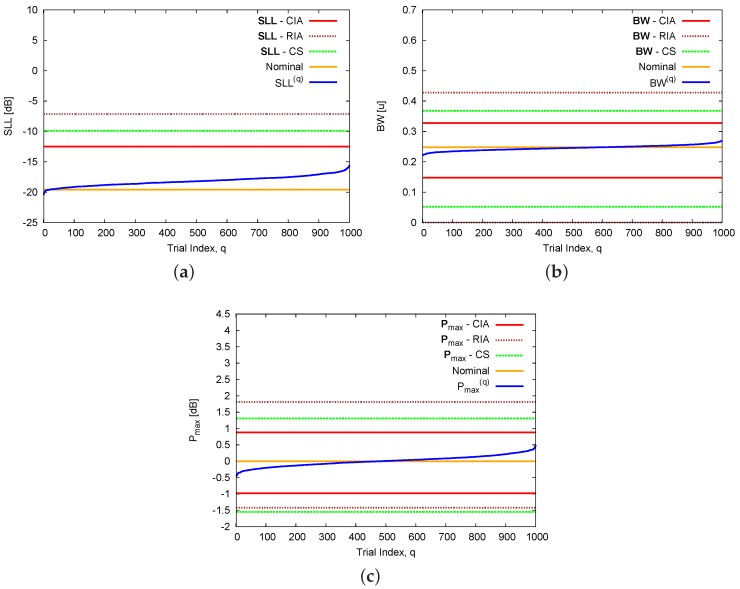
Adjacent Mutual Coupling (N=8, $d=\frac{\lambda }{2}$; Dolph–Chebyshev pattern: $SLL_{ref}=-20\;$ dB)—Nominal values of the pattern indexes and bounds of the intervals (**a**) SLL; (**b**) BW; and (**c**) $P_{max}$ as predicted by the CIA, the RIA , and the CS [16].

The next two test cases deal with excitations uncertainties due to mutual coupling effects. More specifically, the second example is concerned with mutual interactions between physically-adjacent array elements without calibration errors ($c_{n,n}=\gamma_{n}=0$, n=1,...,N). When the coupling coefficients are given in Table 1 (Adjacent Coupling), the bounds of the interval power pattern predicted with the CIA, the RIA, and the CS turn out to be those in Figure 6, while the corresponding interval pattern indexes are reported in Figure 7 and Table 2. By keeping the analysis limited to the comparison between the CIA and the CS results, the RIA bounds being over-estimated, there is a non-negligible improvement with a reduction of more than 2.5dB in the upper bound of the secondary lobes ($SLL_{Sup}^{CS}=-9.88\;$dB *vs.* $SLL_{Sup}^{CIA}=-12.49\;$dB—Table 2 and Figure 7a) also with respect to the previous example when $SLL_{Sup}^{CS}=-15.68\;$dB *vs.* $SLL_{Sup}^{CIA}=-16.60\;$ dB. Moreover, the advantages of using CIA are further highlighted by the reduction of 43% ($\omega^{CIA}\left(\mathrm{BW}\right)=0.180\;\left[u\right]$ *vs.* $\omega^{CS}\left(\mathrm{BW}\right)=0.316\;\left[u\right]$) and 35% ($\omega^{CIA}\left(P_{max}\right)=1.86\;$dB *vs*. $\omega^{CS}\left(P_{max}\right)=2.86\;$dB) of the interval width on the half-power beamwidth (Table 2 – Figure 7b) and the peak power (Table 2 – Figure 7c), respectively.

**Figure 8:**
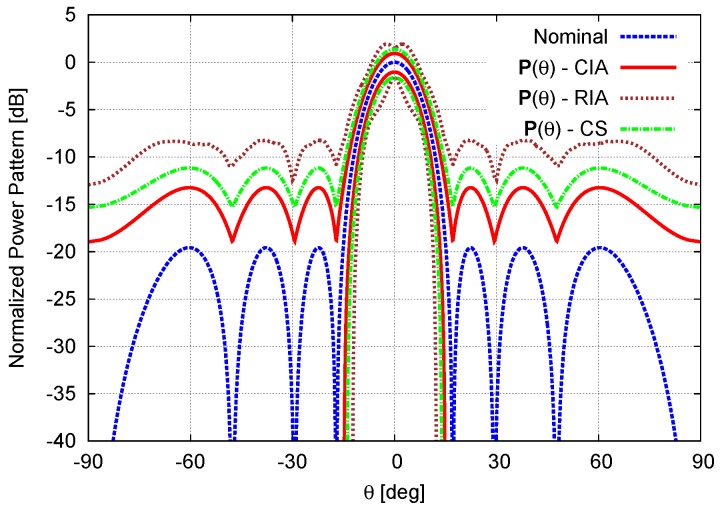
Multiple Mutual Coupling (N=8, $d=\frac{\lambda }{2}$; Dolph–Chebyshev pattern: $SLL_{ref}=-20\;$dB)—Nominal power pattern and interval power pattern bounds predicted by the CIA, the RIA, and the CS [16].

Similar conclusions can be drawn in the third example when multiple coupling effects between non-adjacent elements have also been taken into account. With reference to the coupling coefficients in Table 1 (*Multiple Coupling*), the estimated power pattern bounds as well as the end-points of the pattern features appear to be close to those yielded in the previous example as indicated by Figure 8 and Table 2, respectively.

As far as the computational issues are concerned, the CPU time required by the CIA running on a standard 2.4 GHz laptop with 2 GB of RAM did not exceed $2\times 10^{-2}\;$s thanks to the analytic definition of the $P\left(\theta \right)$ bounds, which does not imply neither numerical integrations but simple arithmetic operations, nor several repeated computations as in Monte Carlo methods.

### 3.2. Prediction Accuracy Evaluation

**Figure 9:**
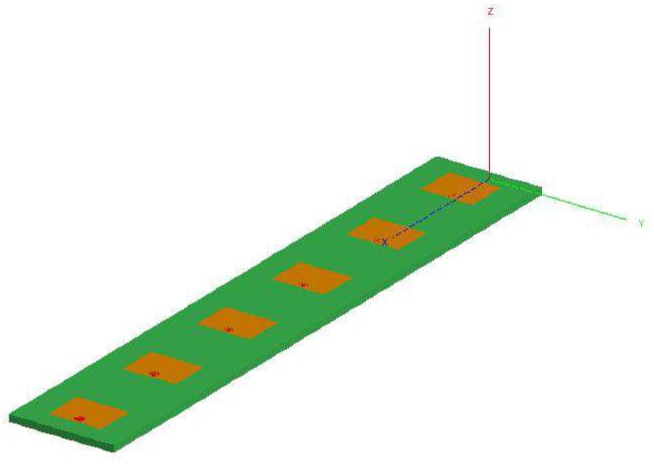
Array Geometry (N=6, $d=\frac{\lambda }{2}$)—Sketch of the linear array of rectangular patches.

The last example is aimed at giving some indications on the profitable use of the proposed CIA-based sensitivity analysis tool for the design of realistic antenna arrays. Towards this end, a linear array of N=6 equally-spaced (d=0.8λ) rectangular (L=0.302λ and W=0.395λ) patches working at f=10.0 GHz and fed with excitations generating a Dolph-Chebyshev pattern with $SLL_{ref}=-30\;$dB [1,3] has been considered (Figure 9). The array elements were placed over a dielectric substrate of total length $L_{sub}=5.5\lambda$ and width $W_{sub}=0.79\lambda$ characterized by a permittivity $\varepsilon_{sub}=2.2$ and thickness h=0.053λ. Concerning the numerical modelling, the presence of real array elements has been taken into account by means of the following element factor [15]

(18) $$EF\left(\theta \right)=\frac{sin\left(\pi hcos\theta \right)cos\left(\pi Wcos\theta \right)}{\pi hcos\theta }$$

then re-defining the field generated by the array as follows

(19) $$AF_{Nom}\left(\theta \right)=EF\left(\theta \right)\times \frac{1}{\chi }\sum_{n=1}^{N}w_{n}e^{jk(n-1)dsin\theta }$$

**Figure 10:**
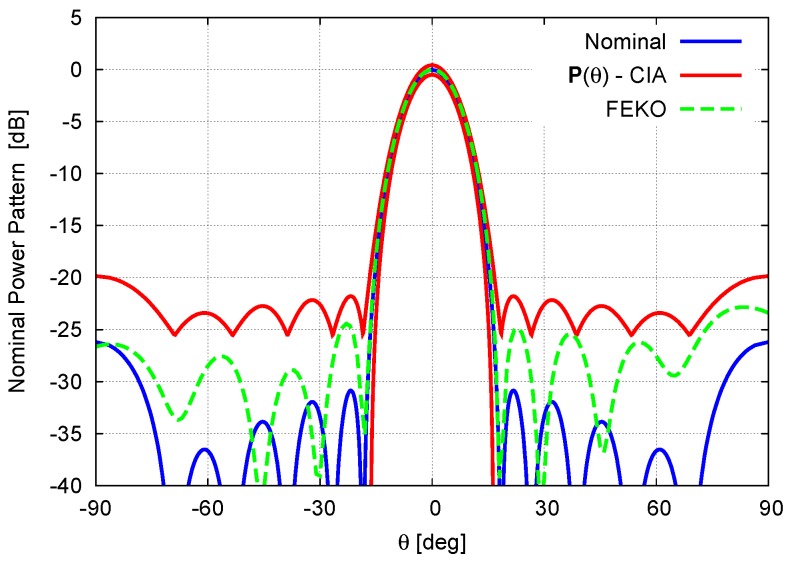
Full-Wave Simulation (N=6, d=0.8λ; Dolph–Chebyshev pattern: $SLL_{ref}=-30\;$dB)—Nominal Equation (19) power pattern, FEKO [27]—computed power pattern, and CIA-predicted power pattern bounds.

Figure 10 shows the nominal (*i.e.*, without mutual coupling, Equation (19)) power pattern, the actual one as computed by FEKO [27], and the CIA-predicted interval power pattern bounds when setting the coupling coefficients to the values provided by the full-wave solver and reported in Table 3. First and foremost, it is worth pointing out that the actual pattern and the nominal one differ in a non-negligible way with a quite evident deterioration of the secondary lobes as well as an overall increment of their levels in the whole sidelobe region. Such an event strongly motivates the use of a suitable sensitivity analysis tool during the array synthesis to *a priori* take into account (and counteract) the undesired effects of uncertainties and errors. On the other hand, the actual pattern is always below the CIA upper bound whatever the observation angle *θ*, but they turn out to be very close in both the mainlobe region and where there are non-negligible radiations (*i.e.*, $P\left(\theta \right)>-30\;$dB). For the sake of completeness, the pattern features of the nominal, actual, and interval solutions are given in Table 4 to further confirm the reliability and potential usefulness of the CIA-based approach.

## 4. Conclusions

An innovative method based on the CIA has been proposed for the sensitivity analysis of the power pattern of linear antenna arrays in the case of calibration errors and mutual coupling effects.

The main contributions of this paper are:

- to the best of the authors’ knowledge, the first time exploitation of the CIA for the sensitivity analysis in antenna arrays when uncertainties and mutual coupling arise in complex (*i.e.*, amplitude and phase) excitations coefficients;
- a compact and efficient definition of complex intervals in terms of their barycenters (*i.e.*, nominal/uncertainty-free values) and radii (*i.e.*, maximum amplitude deviations);
- the definition of analytic power pattern bounds requiring neither the knowledge nor an estimation of the phase deviations or uncertainties, but only based on the value of the nominal array factor and the maximum amplitude error.

From the numerical analysis, it appears that:

- the CIA bounds are accurate and reliable as well as inclusive;
- the CIA-based tool provides a more accurate worst-case prediction of the power pattern tolerances since the CIA bounds turn out to be narrower than those from [16] and the RIA;
- the CIA approach allows one a faithful *a priori* estimation of the behavior of actual power pattern in the high-energy angular regions.

## Figures and Tables

**Table 1:** Calibration Errors, Adjacent and Multiple Mutual Coupling (N=8, $d=\frac{\lambda }{2}$; Dolph–Chebyshev pattern: $SLL_{ref}=-20\;$dB)—Nominal excitations ($\underline{w}=\left\{w_{n}:\;n=1,...,N\right\}$), calibration error coefficients ($c_{n,n}≜\gamma_{n}$, n=1,...,N), and mutual coupling coefficients ($c_{i,j}≜\xi_{i,j}$, i,j=1,...,N, i≠j) [16].

	Nominal Excitations							
*n*	1	2	3	4	5	6	7	8
$w_{n}$	0.0958	0.1060	0.1394	0.1588	0.1588	0.1394	0.1060	0.0958
	Calibration Error							
$\gamma_{n}\left[\%\right]$	2	3	4	5	5	4	3	2
	Adjacent Coupling							
(i,j)	(1,2)	(2,3)	(3,4)	(4,5)	(5,6)	(6,7)	(7,8)	−
$\xi_{i,j}=\xi_{j,i}\;\;\left[\%\right]$	3	5	7	9	7	5	3	−
	Multiple Coupling							
(i,j)	(1,2)	(2,3)	(3,4)	(4,5)	(5,6)	(6,7)	(7,8)	−
$\xi_{i,j}=\xi_{j,i}\;\;\left[\%\right]$	3	5	7	9	7	5	3	−
(i,j)	(1,3)	(2,4)	(3,5)	(4,6)	(5,7)	(6,8)	−	−
$\xi_{i,j}=\xi_{j,i}\;\;\left[\%\right]$	0.2	0.3	0.4	0.5	0.4	0.3	−	−

**Table 2:** Calibration Errors, Adjacent and Multiple Mutual Coupling (N=8, $d=\frac{\lambda }{2}$; Dolph–Chebyshev pattern: $SLL_{ref}=-20\;$dB)—Pattern features of the nominal power pattern and bounds of the interval power pattern features predicted by the CIA, the RIA, and the CS [16].

	*SLL (dB)*	*BW (u = sinθ)*	$P_{max}\left(dB\right)$	Δ
Nominal	−19.58	0.248	0.00	−
	CalibrationError			
CIA	[−23.70;−16.60]	[0.216;0.276]	[−0.33;0.32]	0.1493
CS	[−25.79;−15.68]	[0.204;0.288]	[−0.45;0.43]	0.2050
RIA	[−29.00;−13.62]	[0.192;0.300]	[−0.47;0.56]	0.3487
	AdjacentCoupling			
CIA	[−∞;−12.49]	[0.148;0.328]	[−0.98;0.88]	0.4373
CS	[−∞;−9.88]	[0.052;0.368]	[−1.55;1.31]	0.6893
RIA	[−∞;−7.13]	[0.000;0.428]	[−1.42;1.81]	1.1471
	MultipleCoupling			
CIA	[−∞;−12.20]	[0.140;0.332]	[−1.04;0.93]	0.4619
CS	[−∞;−9.54]	[0.000;0.376]	[−1.63;1.37]	0.7277
RIA	[−∞;−6.71]	[0.000;0.440]	[−1.51;1.91]	1.2225

**Table 3:** Full-Wave Simulation (N=6, d=0.8λ; Dolph–Chebyshev pattern: $SLL_{ref}=-30\;$dB)—Mutual coupling coefficients.

*(i,j)*	(1,2)	(2,3)	(3,4)	(4,5)	(5,6)
$\xi_{i,j}\;\;\left[\%\right]$	3.23	3.76	4.37	3.87	3.46
$\xi_{i,j}\;\;\left[\%\right]$	3.61	3.75	4.34	3.84	3.52

**Table 4:** *Full-Wave Simulation* (N=6, d=0.8λ; Dolph–Chebyshev pattern: $SLL_{ref}=-30\;$dB)—Pattern features of the nominal power pattern and FEKO-computed along with bounds of the interval power pattern features predicted with the CIA.

	*SLL (dB)*	*BW (u = sinθ)*	$P_{\max}$ *(dB)*
Nominal	−26.20	0.236	0.00
Full−Wave	−22.84	0.244	0.00
CIA	[−∞;−19.38]	[0.192;0.276]	[−0.47;0.45]

**Figure B1:**
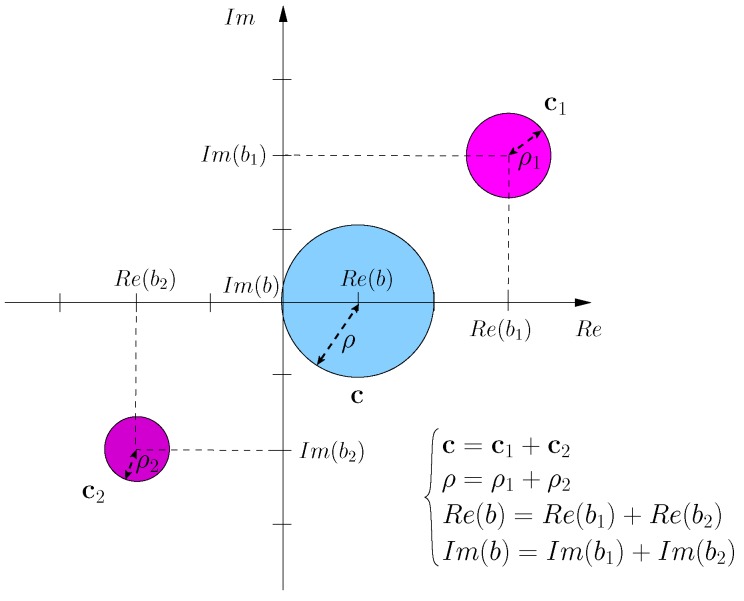
IA-based Approach—Sum of circular intervals.

## Abbreviations

The following abbreviations are used in this manuscript:

CIA: Circular Interval Analysis
CS: Cauchy–Schwartz
IA: Interval Analysis
MC: Mutual Coupling
RIA: Rectangular Interval Analysis

## Appendix A. Circular Interval Definition

A complex circular interval $c=\langle b;\;\rho \rangle$ is defined by the set

(A1) $$c=\left\{a\in C:\;\left|a-b\right|\leq \rho \right\}$$

where C is the set of complex numbers, *b* is the interval barycenter, and *ρ* is the interval radius.

## Appendix B. Circular Interval Arithmetics

The basic operations on circular interval numbers useful for the definition of the circular interval functions of the array factor and power pattern are reported in the following [26]:

### Appendix B.1. Product between Circular Intervals and Complex Numbers

The product between a complex circular interval $c=\langle b;\;\rho \rangle$, of center *b* and radius *ρ*, and a complex number *z* is defined as

(B1) $$z\;c=\langle z\;b;\;\left|z\right|\rho \rangle$$

and the result is still a circular interval centered at zb with radius $\left|z\right|\rho$.

### Appendix B.2. Sum of Circular Intervals

The sum of two complex circular intervals $c_{1}=\langle b_{1};\;\rho_{1}\rangle$ and $c_{2}=\langle b_{2};\;\rho_{2}\rangle$ is equal to (Figure B1):

(B2) $$\begin{array}{ccc} c_{1}+c_{2} & = & \langle b_{1};\;\rho_{1}\rangle +\langle b_{2};\;\rho_{2}\rangle \\ & = & \langle b_{1}+b_{2};\;\rho_{1}+\rho_{2}\rangle \end{array}$$

and the result is a circular interval with center the sum of the interval centers, $b_{1}+b_{2}$, and radius the sum of the interval radii, $\rho_{1}+\rho_{2}$.

### Appendix B.3. Module of a Circular Interval

The module of a circular interval $c=\langle b;\;\rho \rangle$, of center *b* and radius *ρ*, is a real-valued interval defined as in [18]

(B3) $$\left|c\right|=\left[c_{inf};\;c_{sup}\right]$$

$c_{inf}$ and $c_{sup}$ being the lower and the upper bounds of the interval, equal to

(B4) $$c_{inf}=\max\left\{\left|b\right|-\rho ;\;0\right\}$$

and

(B5) $$c_{sup}=\left|b\right|+\rho$$

respectively.
